# Reply to Viner, A.; Ayrey, S. Comment on “Scheepers et al. Comparative Performance Testing of Respirator versus Surgical Mask Using a Water Droplet Spray Model. *Int. J. Environ. Res. Public Health* 2021, *18*, 1599”

**DOI:** 10.3390/ijerph19106296

**Published:** 2022-05-22

**Authors:** Paul T. J. Scheepers, Heiman F. L. Wertheim, Maurice van Dael, Rob Anzion, Henk Jan Holterman, Steven Teerenstra, Martijn de Groot, Andreas Voss, Joost Hopman

**Affiliations:** 1 Department for Health Evidence, Research Laboratory Molecular Epidemiology, Radboudumc, 6500 HB Nijmegen, The Netherlands; maurice.vandael@radboudumc.nl (M.v.D.); rob.anzion@radboudumc.nl (R.A.); 2 Department of Medical Microbiology, Radboudumc, 6500 HB Nijmegen, The Netherlands; heiman.wertheim@radboudumc.nl (H.F.L.W.); andreas.voss@radboudumc.nl (A.V.); joost.hopman@radboudumc.nl (J.H.); 3 Radboudumc Centre for Infectious Diseases, Radboudumc, 6500 HB Nijmegen, The Netherlands; 4 Wageningen Plant Research, Wageningen University and Research, 6700 AA Wageningen, The Netherlands; henkjan.holterman@wur.nl; 5 Department for Health Evidence, Section Biostatistics, Radboudumc, 6500 HB Nijmegen, The Netherlands; steven.teerenstra@radboudumc.nl; 6 Radboudumc REshape Center, Radboudumc, 6500 HB Nijmegen, The Netherlands; martijn.degroot@radboudumc.nl; 7 Department of Medical Microbiology and Infectious Disease, Canisius Wilhelmina Hospital, 6532 SZ Nijmegen, The Netherlands

The response to our publication by Andrew Viner and Stewart Ayrey is much appreciated [1]. The comment addresses the use of the term total inward leakage (TIL), limitations of a dummy head and bioaerosol testing. Below we will respond to each of these points.

We used the concept of TIL and did not refer to the specific method for the measurement of TIL as described in EN149 [2]. We do not agree with the suggestion that this term is exclusively linked to that method. It would not be possible because a surgical mask is not covered by this standard. We see no harm in the use of this term and have clearly indicated that we are using it to compare the performance of a respirator vs. a surgical mask. The concern that the term TIL should be used with reference to the specific method described in EN149 is not realistic. When searching for ‘total inward leakage’ on Google, the first hit refers to a statement by the National Institute of Occupational Safety and Health [3]: “*Total inward leakage (TIL) is an estimate of the performance of a respirator, which is measured as the leakage of contaminants through the filter media and through the face-seal interface and exhalation valve of respiratory protective devices under laboratory conditions. Several test agents have been used to measure TIL in different countries. There is a lack of consensus on the most appropriate test method to measure TIL*”. Therefore, it is not useful to discuss this with respect to its application in EN149:2001 [2]. TIL as a term is valuable in a wider context, i.e., to try to understand how respiratory equipment works, referring to respirators adhering to a specific standard.

Regarding the dummy head, we cannot agree more that a dummy head has many limitations compared to the practice of real users. In our case, we used it to standardize our laboratory technical set-up in order to compare different models and types of protective equipment, which would not be possible with the involvement of human volunteers. We selected the Sheffield dummy head because it is already specified in the EN149+A1:2009 standard [2]. We agree that the lack of ears is a particular limitation of the Sheffield head and that adding ears would be an improvement.

Our research complements findings in real-life based on a large body of evidence for virus infections in healthcare workers, and how these relate to the type of protection used: surgical masks vs. respirators [4,5]. The IIR type of surgical mask is recommended for professionals during a pandemic, according to EN 14683:2019 [6]. In the healthcare sector, respirators (with FFP2 or N95) are recommended for use during aerosol-generating medical procedures. For other tasks, good quality surgical masks provide sufficient protection. Since our paper was published, two additional systematic reviews have analyzed the current collection of studies, including the more recent studies. Again, no observable difference was reported for performances between healthcare workers wearing surgical masks compared to those wearing respirators [7,8]. In the study by Li and co-workers, six randomized clinical trials were included. Only for betacoronavirus, low-quality evidence indicated a better performance for respirators compared to surgical masks. In the most recent update of the WHO recommendation on the use of masks and respirators for health care workers providing care to patients with suspected or confirmed COVID-19 ([9]), it was *“deemed uncertain if respirators are more effective than medical masks in settings without exposure to aerosol-generating medical procedures (AGPs)”.*

The issue of bioaerosols has no direct link to our study as we used a water droplet spray as our model and not a bioaerosol. We did not want to challenge the WHO recommendation of using a respirator in an environment with AGPs [9]. We have an interest in most other clinical settings, where the virus was emitted from the patient through a wide range of bodily fluids (see the figure in our paper). Water droplets have different properties compared to solid particles, are less stable and break up into smaller units due to mechanical forces. Water droplets may reduce in diameter due to water loss by evaporation but retain the same virus load. We believe that this is much better reflected by our fluorescent dye model than by the NaCl model.

We believe that our research contributed to an increased understanding of the use of different types of protective equipment (according to different standards) that are suitable for protection against respiratory infections. Our observations support no higher performance of respirators over surgical masks in a technical set-up simulating transmission by water droplet spray across a wide size range. It is understandable that, technically speaking, the “best possible protection” should be available to all healthcare workers, but there are other aspects to consider as well: regarding the problems that respirators are considered “suffocating”, “uncomfortable” and “difficult to tolerate for long durations” and leading to “facial bruising and abrasions” [10], it is important to also take into consideration aspects of comfort for the wearer.

Perhaps there is one issue we can agree on: standards and terminology are useful for defining and certifying respiratory protective equipment. However, there may not always be a good match between these standards and real-life settings in the changing reality of a pandemic. It is not uncommon that there are discrepancies between pre- and post-market evaluations of technology that need to be addressed to continuously improve existing technologies [11].
